# Effects of physical stress in alpine skiing on psychological, physiological, and biomechanical parameters: An individual approach

**DOI:** 10.3389/fspor.2022.971137

**Published:** 2022-10-10

**Authors:** Thomas Finkenzeller, Tim Burberg, Stefan Kranzinger, Eric Harbour, Cory Snyder, Sabine Würth, Günter Amesberger

**Affiliations:** ^1^Department of Sport and Exercise Science, University of Salzburg, Salzburg, Austria; ^2^Salzburg Research Forschungsgesellschaft mbH, Salzburg, Austria; ^3^Red Bull Athlete Performance Center, Thalgau, Austria

**Keywords:** individuality, holistic approach, physical load, fatigue processes, breathing pattern, experienced male skier, wearable sensors

## Abstract

Alpine skiing is an attractive winter sport that often includes mental and physical demands. Since skiing is often done for several hours, fatigue processes occur that might lead to action errors associated with a higher risk of accidents and injuries. The aim of this study was to investigate the timing of changes in subjective, physiological, and biomechanical parameters during a physically demanding, standardized, non-competitive alpine skiing session. A group of 22 experienced male skiers carried out 10 runs, each lasting between 150 and 180 s, at a turn rate of 80 turns per minute with their best skiing technique. Immediately after the run, skiers reported ratings of fatigue, and other affective states. During skiing, breathing pattern and biomechanical data of the ski turns as radial force, turn duration, edge angle symmetry, and a composed motion quality score were recorded. Analyses of variances on skiers showing signs of fatigue (*n* =16) revealed that only the subjective data changed significantly over time: fatigue and worry increased, vitality and calm decreased. Subsequently, individual change points analyses were computed to localize abrupt distribution or statistical changes in time series data. For some skiers, abrupt changes at certain runs in physiological and/or biomechanical parameters were observed in addition to subjective data. The results show general effects in subjective data, and individual fatigue-related patterns concerning the onset of changes in subjective, physiological, and biomechanical parameters. Individuality of response to fatigue should be considered when studying indicators of fatigue data. Based on the general effects in subjective data, it is concluded that focusing on self-regulation and self-awareness may play a key role, as subjective variables have been shown generally sensitive to the physical stress in alpine skiing. In the future, customized algorithms that indicate the onset of fatigue could be developed to improve alpine skiers' self-awareness and self-regulation, potentially leading to fewer action errors.

## Introduction

Alpine skiing is a highly attractive sport for many people, often practiced only a few days a year, but then for several hours. The combination of high motivation, comparatively little specific training, and an intensive acute physical and mental stress can lead to delayed or insufficient processing of fatigue. Action psychological approaches postulate that fatigue is a central predictor of action errors. The latter, in turn, are directly causally related to the occurrence of accidents and injuries (1, 2). Although some findings from occupational psychology indicate that work overload leads to increased susceptibility to errors and inaccuracy or to poorer perception of external stimuli (3, 4), there are few studies that have addressed changes in internal processes at the emotional, physiological, and coordinative level. Especially in sports activities with high coordinative and motor demands such as alpine skiing, it is critical to study processes that are associated with physical load against the background of the three systems approach (5). This approach, which originated in psychotherapy research, assumes that an emotional reaction is composed of subjective experience, physiological reactions, and overt behavior. Applying this approach to sport science research has the advantage that complex phenomena such as the effects of physical and mental stress associated with changes in fatigue state on self-regulatory processes can be studied holistically (6). The scientific findings of changes in psychological, physiological, and coordinative-motor processes could contribute to enhanced understanding of action errors in alpine skiing.

To date, few studies have been conducted with recreational alpine skiers analyzing short- and long-term changes in psychological, physiological, and biomechanical parameters. Most studies addressed exclusively physiological parameters such as heart rate and lactate (7, 8), or changes in muscle force (9) and muscle activity (9–11). To our knowledge, only one study has examined how psychological states change over three measurements in a skiing session of 3.5 h in elderly male and female recreational skiers (*M*_age_ = 63 ± 6 years). Mood states were assessed 0.5, 1.5–2, and 3.5 h after skiing began. In contrast to our study, the participants were asked to ski in their usual skiing style, and they could take a break whenever they wanted. Minor significant increases of less than one on a 11-point Likert scale were observed for fatigue, sociability, and happiness, while no changes were found in the readiness for the activity, concentration, self-confidence, nervousness, anger, or worry (7). Therefore, for this study, we decided to standardize the physical task to elicit detectable sensations of fatigue with accompanying subjective, physiological, and motor-coordinative changes. A physiological approach was used by Seifert et al. (8) who investigated the relationship and predictors of common fatigue indices during recreational skiing in young females (*M*_age_ = 22.7 ± 4 years) during 3 h of skiing. The length of the turns was administered by a standardized corridor, and they were instructed to maintain similar finishing times. Notably, it was found that heart rate does not appear to respond to fatigue (8), perhaps due to the many external influences during a day of skiing. Thus, we selected breathing as physiological indicator in this study, which was recently shown to be a sensitive indicator of signs of fatigue (12). Furthermore, there is empirical evidence that recreational skiing for 4 h is associated with a prolonged (at least 24 h) decrease in eccentric quadriceps and hamstring strength (9). This is in line with findings on electromyographic activity during a 3.5 h alpine skiing session in recreational female skiers indicating a significant decrease in the frequency content of the EMG signal with highest effects for the M. rectus femoris of the outside leg (10). In summary, there is little empirical evidence available regarding the time course of changes in subjective states, physiological, and motor-coordinative behavior throughout a typical skiing day.

It is reasonable to assume that with continued alpine skiing, changes in sensation of fatigue and related physiological parameters, as well as the quality of the skiing, will change. Determining the time course of these changes is necessary to gain further insight in the mechanisms involved in fatigue. In general, the intensity, type, and duration of a physical activity causes adaptions within the body (i.e., biochemical processes, breathing pattern) to maintain the performance, which in turn leads to changes on the central nervous system (13). In addition to these changes, the sensation of fatigue may change depending on physical exertion, training status (14), and psychological skills (15), which subsequently influences adjustments of the exercise strategy. Recently, a framework of fatigue proposed by Enoka and Duchateau distinguishes fatigability from fatigue. Fatigability was defined as an objective change in motor or physical performance, whereas fatigue describes a subjective sensation (16). In their work, fatigue is defined as “a disabling symptom in which physical and cognitive function is limited by interactions between performance fatigability and perceived fatigability”, p. 2228. Accordingly, fatigue can only be determined by self-reports, which is confounded by an individual interpretation of relevant physiological and psychological changes. The sensation of fatigue depends on conscious and unconscious processes, and is unique depending on individual experiences (17). Hence, an individual approach is necessary to explore the complex relationship between fatigue and fatigability, which is considered both dependent and independent and implies many possibilities for fatigue processes (18). This framework is considered in the present study, with a central focus on the assessment of fatigue. The rate of fatigue scale (18) provides a single item questionnaire that can be easily used after each run to obtain reliable self-reporting on changes in fatigue over time. To detect broader, holistic signs of fatigue, affective well-being (subjective level), breathing patterns as indicators of perceived fatigability (physiological level), and biomechanical parameters of the skiing turn (overt behavior) as indicators of performance fatigability were measured. The measurement of these data in the context of a field study is possible thanks to recent technological developments that allow the recording of respiratory activity (19, 20) and biomechanical parameters relevant to alpine skiing (21–23) over several hours with sufficient accuracy and minimal interference to performance. It must be pointed out that, unlike heart rate, breathing pattern is highly sensitive to exercise-induced physiological processes (12, 24) including fatigue during alpine skiing (25). Finally, in addition to a general approach, it was prioritized an individual-focused statistical approach to carefully parse the relationship of fatigue, perceived fatigability, and performance fatigability between and within skiers.

The aim of this study was to investigate the timing of changes in subjective, physiological, and biomechanical parameters during a physically demanding alpine skiing session. To analyze general and individual changes relative to the phenomenon of fatigue two different statistical methods were used. We hypothesized that changes of sensations in fatigue would change individually with increasing duration of skiing, which would be associated with individual patterns of breathing and of the quality of the ski turn.

## Methods

### Participants

A sample of 22 experienced male alpine skiers participated in this study. Participants indicated that they have been regularly skiing for at least 10 years and started skiing during childhood (*M* = 4.0 years, *SD* = 2.2). Additionally, participants were asked to confirm beforehand whether they feel confident in adhering to an acoustically instructed turn frequency of 80 turns per minute. Table 1 shows age, anthropometric, and data on the fitness level of the skiers. The reasons for the selection of this sample were, first, to ensure that skiers can perform the task accurately, second, from an ethical perspective, it was important that the risk of injury was as low as possible, and third, to have a homogeneous sample because, for example, the subjective experience of women might differ from that of men. There were no falls during the entire test period. Participants confirmed that they were healthy and did not suffer from any neurological disorders or cardiovascular diseases. In the morning of the test day, the subjects and the investigators had to present a negative COVID-19 rapid antigen test. A FFP2 mask had to be worn on arrival and departure, when falling below a minimum distance of one meter and during the gondola ride. Furthermore, they were instructed to get enough sleep, drink sufficiently, and avoid hard training sessions the day before the skiing session, as well as to abstain from caffeine 2 h before starting the runs. The local ethics committee approved the study protocol. Each participant signed an informed consent.

**Table 1:** Sample characteristics (*n* = 22).

		***M***	***SD***	**Range**
Age (years)		26.9	5.4	20–45
Body weight (kg)		76.2	6.9	64.0–88.6
Body height (cm)		178.2	6.9	164.0–189.0
Age when started skiing (years)		3.98	2.2	2–10
Single-leg isometric *F*_max_ (N)	Right	1830.4	351.1	1362.7–2639.9
	Left	1751.7	311.5	1202.4–2300.8
Single-leg isometric *F*_expl_ (kN/s)	Right	45.8	12.9	31.9–74.4
	Left	41.6	12.7	10.2–74.4
VO_2_peak (ml/min/kg)		50.6	6.6	34.6–62.6

### Design and procedure

All data collection on the slope took place from 16.02.2021 to 06.03.2021 in Schladming, Austria. Snow conditions were mostly freshly groomed and grippy at the beginning of a testing day turning soft during the day. The weather conditions were sunny to partly cloudy without condensation. Temperature throughout the testing days was consistently between −2 and +4°C (*M* = 2.00, *SD* = 2.17) in the mornings, to −1 and +12°C (*M* = 3.75, *SD* = 3.77) at the end of the testing session. Since this was a north-facing slope, the effects on slope quality were very small, as indicated by biomechanical findings on ski turns (see **Figure 4**). Conditions changed little, if at all, across each person's 10 runs. Furthermore, because the tests took place during the Corona pandemic, very few people used the slope. One day before the alpine skiing experiment (*t*_0_), participants were informed about organizational issues and the study procedure via video conference (WebEx, Webex by Cisco, Milpitas, CA, USA). Four weeks later, a broad physical fitness profile was collected including a single-leg isometric strength test and a measurement of peak oxygen uptake (VO_2_peak). These measurements are used only to describe the fitness level of the skiers (see Table 1).

In this study, we chose a standardized protocol to ensure that we induced detectable sensations of fatigue and a comparable physical load. A preliminary study was conducted to determine the optimal number of runs that would produce a change in fatigue level of at least five points on a 11-point Likert scale and, at best, result in a state of totally fatigued. Accordingly, participants were instructed to perform 10 alpine skiing runs on a red slope [corresponding to Austrian Standards Institute (ASI); ÖNORM S 4611] at an Austrian ski resort (Planai, Schladming, altitude: 1,830–1,350 m, length: 2,200 m). All participants were provided with a Hexoskin (HX) smart shirt (Carré Technologies Inc., Montreal, Canada), which they wore throughout the experiment, and custom instrumented ski boots (Atomic Hawx Ultra 130, Atomic Austria GmbH, Altenmarkt, Austria). Participants completed the runs with their own skis. Prior to skiing, after five and ten runs, the participants completed a computerized cognitive task of around 5 min followed by a 5-min measurement of heart rate variability (HRV) at rest in a room of the cable car company at the middle station of the gondola at 1,350 m altitude. The data on cognitive performance and HRV are not focus of this article and therefore are not reported.

**Figure 1:**
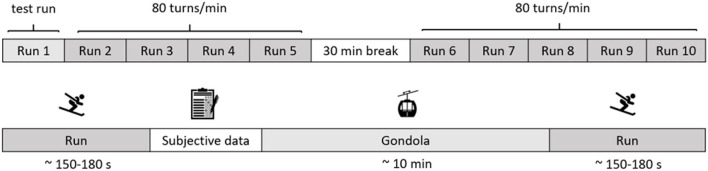
Design and procedure of the alpine skiing study. The first line provides an overall view of the procedure. The second line presents the procedure of within one run to the following run.

After a standardized warm-up guided by a certified skiing instructor, participants were asked to descend the slope while adhering to the prescribed turn frequency of 80 turns per minute. The turn frequency was administered with a smartphone application (Metronome Beats, Stonekick, London, UK) and in-ear headphones. Participants were to perform the maximum possible turn size while maintaining the set turn rhythm. Additionally, they were instructed to ski as much as possible using the carving turns as to demonstrate their “best possible” skiing technique in all runs. The first run served as a familiarization run to get used to the skiing slope and the pre-set turn frequency. During the runs, participants were followed by a skiing instructor who recorded their skiing performance using a handheld video camera. Immediately after the run, participants reported on their affective well-being. The experimental runs lasted between 150 and 180 s and the ride in the gondola lasted about 10 min. There was a break between the fifth and the sixth run of 30 min, during which participants were allowed to have a snack and to drink water (see Figure 1).

### Materials

#### Subjective psychological data

Fatigue, the sensation that should be directly affected by the alpine skiing load, was assessed using the rate of fatigue scale (18). Against the background of the circumplex model of affect (26, 27), three further states of affective well-being were selected. The expressions in the dimensions of arousal (high/low) and valence (positive/negative) are considered by the states of vitality (high arousal and positive valence), fatigue (low arousal and negative valence), calm (low arousal and positive valence), and worry (high arousal and negative valence). Participants were asked to rate their state of vitality (28), fatigue (18), worry, and calm on an 11-point Likert scale ranging from 0 (not true at all) to 10 (totally true) beginning with “At this moment I feel….” The questions were asked by the accompanying ski instructor immediately after each run and the answers were recorded on a piece of paper.

#### Physiological data

Participants wore a HX shirt in all trials to gather respiration (dual thoracic and abdominal stretch sensors, 2 channel respiratory inductance plethysmography, 16-bit, 128 Hz) and heart rate (1 Ch ECG, 12-bit) data. Raw data were extracted via the HxServices application and the Hexoskin Online Dashboard. Breathing pattern was calculated with the algorithm of Harbour et al. (19) via MATLAB R2021a software (The MathWorks Inc., Natick, USA). Breathing rate (five-breath rolling average; breaths per minute) and depth (composite thoracic + abdominal amplitude; arbitrary units) was summarized on a per-run basis using average (mean), variability (coefficient of variation), and slope (between first 30 s and last 30 s average). Complete data sets are available for 12 subjects. In addition to the six participants who are excluded in the analyses on the subjective level, data from four skiers had to be excluded due to bad signal quality.

#### Biomechanical data

Two IMU's mounted to the upper posterior cuff of each ski boot measured 3D ski-boot acceleration and angular velocity signals during each skiing run (Movesense LSM6DSL, 2.5 × 3 × 0.83 mm, ±16 g and ±1,000 dps full scale resolution, ST Microelectronics, Amsterdam, Netherlands). The analog signals were sampled at 833 Hz, filtered, and transmitted to a custom smartphone application for storage and post processing at 54 Hz. For further information regarding the wearable system, see Martínez et al. (22).

The turns were detected using the algorithm proposed previously by Martínez et al. (29, 30). The procedure utilized the mean angular velocity about the Z-axis (roll axis, pointing anteriorly) recorded from IMU's mounted on the upper posterior cuff of each ski boot. According to the inverted pendulum model frequently applied to alpine skiing, the time point of maximum angular velocity occurs when the pendulum is in the neutral position, or during the brief period between turns where the skier is not skiing, termed the turn switch. The algorithm uses a series of filters to first identify possible turn switch points and remove outliers, and second to “fine-tune” the identified turn switch points to find the most precise time point. Data were processed according to the extended activity recognition chain proposed by Brunauer et al. (31), where turns were segmented (29), and time normalized kinematic parameters including edge angle, radial force, speed, and edge angle symmetry were calculated for each turn (21). Based on these variables, turns were classified as carving, drifting, or snowplow (32), and finally assigned a quality score based on the motion quality algorithm (23). The last 30 turns of the runs two to ten were segmented (altitude ~1,572–1,350 m) and an average over these turns was calculated. Four biomechanical indicators of motor-coordinative behavior were preselected so that the same number of variables were included in the change point detection analyses. The choice was based on theoretical considerations (standard deviation of turn duration reflecting the stability of turn timing) and effect sizes obtained by Kendall's W, selecting the parameters with the highest effect sizes (see **Table 3**). Accordingly, *turn duration, SD, edge angle symmetry, mean, radial force, mean*, and *motion quality score, mean* were determined (see Table 2). For completeness, descriptive and analytical statistics for the remaining variables are reported within the Supplementary materials (see Tables 3, 4). Due to data loss or synchronization problems with Bluetooth data, only complete data sets with no data-loss of 10 participants were considered eligible for analysis. To analyze comparable data sets, only data sets of participants who performed exactly 10 runs with no missing runs were included in the analysis.

**Table 2:** Overview of the selected aiming variables.

**Level**	**Variables**	**Description**
Subjective level	Vitality	Scale from 0 to 10
	Fatigue	Scale from 0 to 10
	Worry	Scale from 0 to 10
	Calm	Scale from 0 to 10
Psychophysiological level	Breathing rate, mean	Respiratory frequency (breaths per minute)
	Breathing rate, cv	BR variability; quotient of standard deviation and mean BR (%)
	Amplitude, mean	Breathing depth; composite sum of thoracic and abdominal RIP amplitudes (arbitrary units)
	Breathing rate, slope	Slope difference between first 30 s mean BR and last 30 s within one run
Biomechanical level	Radial force, mean	Mean value of time normalized radial force (g)
	Turn duration, SD	Standard deviation of the turn duration (s)
	Edge angle symmetry, mean	Mean value of the differences in edge angle of both skis (°)
	Motion quality score, mean	Score consisting of edge angle, edge angle symmetry, and radial force, score from 0 to 10 (arbitrary units)

#### Determination of participant's fitness level

##### Maximum voluntary and explosive single-leg isometric strength

The assessment of maximal voluntary and explosive strength was carried out on both legs separately in the leg-press position using a customized apparatus featuring a force plate. The knee angle was fixed at an angle of 100°. Signals were amplified by a DMCplus device (HBM, Darmstadt, Germany) and recorded with LabVIEW 6.1 software (National Instruments, Austin, USA). Maximum voluntary strength (*F*_max_) was determined as the maximum value of force distribution and explosive strength (*F*_expl_) as maximum force increase.

##### Peak oxygen uptake (VO_2_peak)

A ramp incremental cycling protocol (LC7TT, Monark Exercise, Vansbro, Sweden) was applied to assess VO_2_peak via a breath-by-breath spiroergometry system (ZAN600 Spiroergometrie, ZAN Austria e.U., Dietach, Austria). A 5-min warm-up interval at 70 W was followed by a load increase of 30 W per minute until exhaustion. Participants were instructed to maintain a cadence of about 80 rpm throughout the ramp test. VO_2_peak was determined by calculating centered moving averages (15-breaths window) of breath-by-breath data during load increase. The “highest” moving average value was used as VO_2_peak (33).

#### Statistical analyses

For the analyses of the subjective data, only participants were selected who completed 10 runs and demonstrated a change in fatigue of five points or more between the baseline (fatigue at rest at the beginning) and fatigue after the last run. This was to ensure that only participants who had a substantial change in fatigue of at least half the scale included in the analyses. Based on these criteria, four participants were excluded because they were totally fatigued after less than 10 runs, one participant showed too little change in fatigue (change of three points), and one participant scored calm and vitality with 10, and worry with zero at all measurements indicating that the scale was not understood. Thus, subjective data from 16 skiers were available for analyses. The sample size of physiological (*n* = 12) and biomechanical data (*n* = 10) is smaller due to movement artifacts and technical problems. For details see section Materials. Complete data sets including subjective, physiological, and biomechanical data are available for seven subjects.

The holistic approach taken in this study results in many dependent variables, which are summarized in Table 2. Two statistical approaches are used in this study. First, non-parametric univariate repeated measure comparisons (Friedman's ANOVA) were applied to get first insights about changes in subjective, physiological, and biomechanical parameters over time for the whole group. The familiarization run was not considered in the analyses. Shapiro-Wilk tests revealed significant deviation from normality for some runs for all parameters except breathing rate (mean), *breathing depth* (amplitude, mean), and the *motion quality score*. Computed effect sizes of Kendall's *W* were classified in small (*W* ≥ 0.1 and < 0.3), medium (*W* ≥ 0.3 and < 0.5), and large effects (*W* > 0.5) in accordance to Cohen (34). Skewness and kurtosis for all parameters are reported in the Supplementary materials. The significance level was set at *p* = 0.05 for each data level with Bonferroni correction for the number of variables per data level to avoid the accumulation of alpha errors. If significant changes were found, rank signed *post-hoc* tests were computed according to Dunn (35) and finally Bonferroni corrected. R Package *rstatix* was used for non-parametric univariate repeated measure comparisons and *post-hoc* tests (36).

In a second step, change points were computed to consider the individual changes within the subjective, physiological, and biomechanical data level separately and across all three data levels. The aim of change point detection algorithms is to localize abrupt distribution changes or changes of statistical properties in time series data (37, 38). To detect change points in the multivariate time series data, we chose a non-parametric estimation of the number of change points and their position of occurrence (38). In detail, the estimation is based on hierarchical clustering and a divisive algorithm. With this divisive method, no distribution assumptions had to be made in advance and neither prior knowledge of the underlying distribution family nor additional analysis is required to find distributional changes in multivariate time series data. The E-Divisive algorithm (38) can determine the number of change points and their location simultaneously. The definition of the number of change points is data driven and has not to be predefined. It uses a bisection procedure (39) and a divergence measure based on the concept of Euclidean distances and the work of Szekely and Rizzo (40).

For the analyses, change point detection via the R package *ecp* (41) and the *e.divisive* function was applied. We follow the explanation of the ecp package (41) to explain the general procedure of the algorithm. In the hierarchical divisive estimation, multiple change points are estimated by iteratively applying a single change point location procedure. The position of the change point is re-estimated at each iteration by dividing an existing segment. The flow of this method can thus be represented as a binary tree, where the root nodes represent the case where there are no change points and the entire time series is contained. The non-root nodes are a copy of the parent node or a new segment. The latter is created by adding a change point to its parent. A permutation test is used to perform the statistical significance of the estimated change points. This is necessary because the distribution of the test statistic results from the distributions of the observations, which in our case is not known (41).

## Results

### Non-parametric univariate repeated measure comparisons (Friedman's ANOVA)

#### Subjective parameters

**Figure 2:**
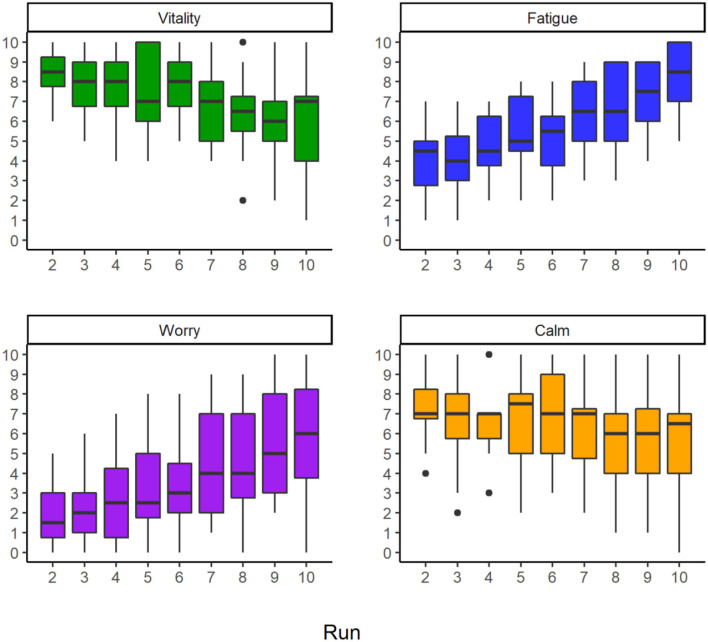
Boxplots including outliers (1.5 times of the interquartile range) indicated as black dots of subjective parameters (*n* = 16) per run.

Non-parametric repeated measure comparisons revealed significant differences between runs for all subjective parameters (see Table 3). *Vitality* gradually decreased significantly, while *fatigue* and *worry* increased significantly with continuation of alpine skiing. *Calm* changed significantly and showed a slight decrease from run two to run five, partially recovered at run six, slightly decreased again, and remained relatively stable from run seven to run ten (see Figure 2 and Supplementary Table 1).

**Table 3:** Friedman's ANOVA for subjective, physiological, and biomechanical parameters.

	**Friedman' s ANOVA**		
	**χ^2^**	***p***	***W***
**Subjective parameters (*****n*** **= 16)**			
Vitality	62.2	<0.001^†^	0.49
Fatigue	94.5	<0.001^†^	0.74
Worry	83.6	<0.001^†^	0.65
Calm	33.6	<0.001^†^	0.26
**Physiological parameters (*****n*** **=12)**			
Breathing rate, mean (bpm)	8.3	1.0^†^	0.09
Breathing rate, cv (%)	12.4	0.53^†^	0.13
Breathing rate, slope	5.2	1.0^†^	0.05
Breathing depth amplitude, mean (au)	12.9	0.46^†^	0.13
**Biomechanical parameters (*****n*** **= 10)**			
Turn duration, SD (s)	16.6	0.14^†^	0.20
Edge angle symmetry, mean (°)	7.4	1.0^†^	0.09
Radial force, mean (g)	8.1	1.0^†^	0.10
Motion quality score, mean	14.0	0.32^†^	0.18

au, arbitrary units; cv, coefficient of variation; W, Kendall's W.

† Bonferroni corrected.

For *fatigue*, significant Bonferroni corrected Dunn *post-hoc* findings were found between run two and runs eight, nine, and ten (*p*_Run2vs.Run8_ = 0.03; *p*_Run2vs.Run9_ <0.01; *p*_Run2vs.Run10_ < 0.001), between run three and runs nine and ten (*p*_Run3vs.Run9_ < 0.01; *p*_Run3vs.Run10_ < 0.001), run four and run ten (*p*_Run4vs.Run10_ < 0.01) as well as run six and run ten (*p*_Run6vs.Run10_ = 0.02). *Post-hoc* tests for *vitality* showed a significant decrease between run two and run nine (*p*_Run2vsRun9_ = 0.04). *Worry* increased significantly between run two and runs nine and ten (*p*_Run2vsRun9_ = 0.008; *p*_Run2vsRun10_ = 0.008) as well as run three and runs nine and ten (*p*_Run3vsRun9_ = 0.022; *p*_Run3vsRun10_ < 0.020). No significant *post-hoc* differences between runs were determined for *calm*.

#### Physiological parameters

**Figure 3:**
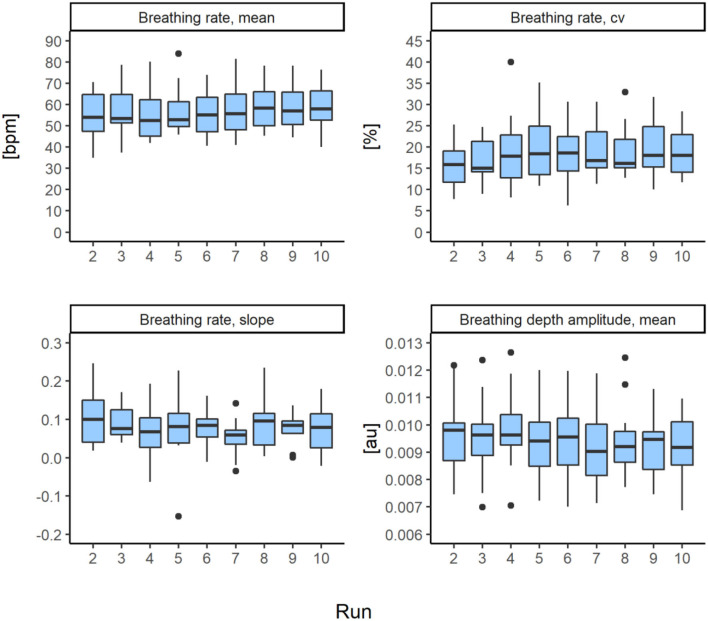
Boxplots including outliers (1.5 times of the interquartile range) indicated as black dots of physiological parameters (*n* = 12) per run.

Non-parametric repeated measure comparisons showed no significant differences between runs in *breathing rate mean, breathing rate slope mean, breathing rate cv*, and *breathing depth amplitude* (see Table 3). For details on descriptive statistics see Figure 3 and Supplementary Table 2.

#### Biomechanical parameters

**Figure 4:**
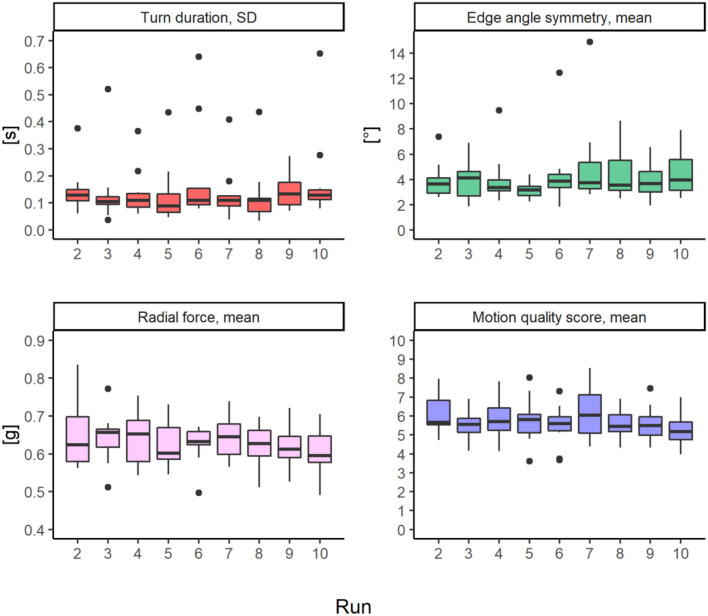
Boxplots including outliers (1.5 times of the interquartile range) indicated as black dots of biomechanical parameters (*n* = 10) per run.

*Turn duration SD, edge angle symmetry, radial force*, and *motion quality score* revealed no significant change over the runs (see Table 3). For details see Figure 4 and Supplementary Table 2.

### Change point detection

**Figure 5:**
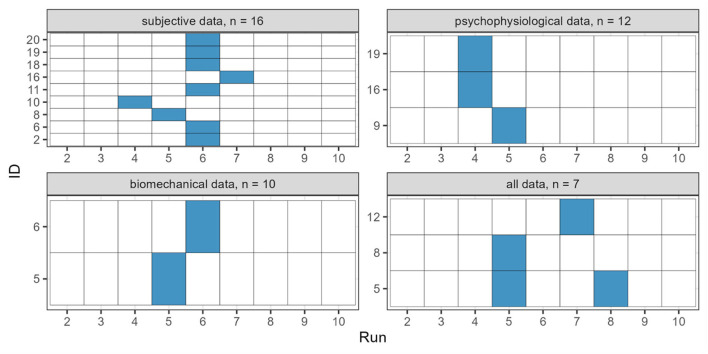
Detected change points in the time series; note that the number of participants, as indicated in the header, vary by data level; Subjective data consist of: fatigue, vitality, worry, and calm; psychophysiological data consist of: breathing rate, mean, breathing rate, cv, breathing rate, slope, and breathing depth amplitude, mean; biomechanical data consist of: turn duration, SD, edge angle symmetry, mean, radial force, mean, and motion quality score, mean.

Figure 5 illustrates the detected change points in the time series for each data level separately. The x-axis shows the number of the run, and the y-axis represents the participant's code. If a change point has occurred, the rectangle is filled in blue for the respective run. The number of analyzed participants is indicated in the title bar of each sub-figure and ranges from 10 for biomechanical data to 16 for subjective data. Complete data sets were available from seven participants. In the subjective data, change points were obtained in nine of the sixteen participants. Six of the nine change points were noted immediately after the sixth run, which was preceded by a half-hour break. For the physiological data, change points were obtained in three out of twelve participants, while for the biomechanical data, two out of ten participants showed change points. Analyzing all three data level together, change points were found in three out of seven participants. About two-thirds of the observed change points occurred after the fifth and sixth run. The individual changes for each participant are reported within the Supplementary materials (see Figures 1–4). Individual patterns emerge in the combination of subjective, physiological, and biomechanical as well as when including all parameters that led to the detected change point.

**Figure 6:**
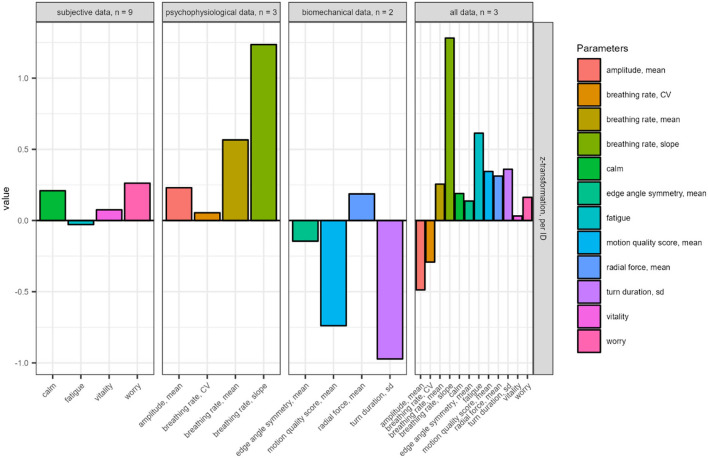
Mean absolute changes of the z-transformed parameters at the change point.

The mean absolute changes of the z-transformed parameters after a change point are shown in Figure 6. For example, if a change point was observed after the sixth run, the absolute change compared to the fifth run is shown. Subsequently, the mean absolute change over all detected change points is calculated and shown in Figure 6 for each data level and for all variables. On average, the subjective data increased in *calm, vitality*, and *worry*, while *fatigue* remained relatively stable. Physiological data showed on average a remarkable increase in *breathing rate slope* and *breathing rate mean*. *Breathing depth amplitude mean* increased moderately and *breathing rate cv* was relatively stable. On the biomechanical level, the *motion quality score, turn duration SD*, and *edge angle symmetry mean* decreased on average, while the *radial force mean* increased. Change point detection on all parameters showed on average an increase of more than one standard deviation in the *slope of the breathing rate*, an increase of about half a standard deviation in *fatigue* and a decrease of about half a standard deviation in *breathing depth amplitude mean*.

## Discussion

This study examined changes in alpine skiing behavior against the background of the three systems approach (5) considering data on the subjective, physiological, and biomechanical levels. The aim of this holistic approach was to gain more knowledge on the processes that are associated with skiing-induced fatigue. Fatigue was considered as an individual sensation arising from the interpretation of perceived and performance-related factors (16). By combining a general statistical approach with an individual approach, general and individual trends were found.

### Overall effects

#### Subjective level

In general, it was found that the study protocol resulted in a significant increase of *fatigue* with a large effect size (*W* = 0.74). In addition, the alpine skiing session led to a significant decrease in *vitality* (*W* = 0.49) and *calm* (*W* = 0.26), while *worry* increased significantly (*W* = 0.65). According to circumplex of affect (26), this can be interpreted that arousal decreases, and valence develops negatively by the alpine skiing session. The findings are not in line with a previous study, which reported an increase of positive valence despite an increase of fatigue in recreational skiers (7). The performance-oriented task of completing 80 turns per minute with the best skiing technique over nine runs may have led to doubts about not being able to fulfill the task, reflected in an observed sharp decline in *worry*. Overall, there is a small number of published studies of the effects on affective well-being during sports. Nevertheless, this study has contributed to this important topic by demonstrating that a standardized alpine skiing session leads to large changes in *fatigue*, which is accompanied by moderate to large changes in *vitality, calm*, and *worry*. The interrelation between these subdimensions was beyond the scope of this research but should be investigated in the future.

#### Physiological level

Physiological metrics exhibited non-significant, small effect sizes for pooled group data. However, about half of the skiers experienced an increased *breathing rate* across the skiing session, which was moderately correlated with fatigue (*r* = 0.56, *p* = 0.02). In a recent study on this data set, it was reported that some of the skiers did not increase *breathing* rate were performing some degree of Locomotor-respiratory coupling [breath and turn entrainment (25)]. Breathing coupled to fixed-tempo turning (as prescribed in this study) inherently leads to a stable *breathing rate*. It is unknown whether the coupling was done consciously or unconsciously; it could be driven by the rhythmic sound cues used in this study or biomechanical influences such as those present in running (42). Thus, future studies should investigate the development of breathing patterns during prolonged skiing when the turn frequency is self-selected.

#### Biomechanical level

Contrary to our expectation, no significant changes were found in biomechanical data indicating motor-coordinative behavior. Skiers maintained stable turn quality as estimated by the biomechanical parameters, can be maintained despite a high degree of fatigue. This contradicts previous studies analyzing EMG patterns and observations of the skiing technique (10, 11). Possibly, the experienced skiers have well-developed motor compensation strategies to accomplish the task of skiing 80 turns per minute with their best technique. There is empirical evidence that fatigue is associated with compensatory sensorimotor processes that set high cognitive load on the skier, possibly leading to a higher risk of action errors (43), an aspect that should be considered in future research. The fact that experienced skiers subjectively perceive a clear fatigue but do not show any deterioration in terms of skiing technique suggests that they are able to compensate for fatigue very well, presumably by making clever adjustments that allow them to still make a good turn. Whether this leads to an increased risk cannot be stated based on these data. Furthermore, we propose that investigations upon movement quality over long time periods should use more sophisticated methods than those used in this study to gain more knowledge on kinetic and kinematic changes.

### Individual effects

#### Change points in the time series

Individual analyses were calculated to identify change points in the time data. While skiers had a diverse timing of change points in their subjective data, the sixth run was the most common significant change point. Notably, the relationship of the subjective variables to each other appeared to be unique between individuals (see Supplementary Figures 1–4). However, it should be mentioned that in seven out of sixteen participants no change point was detected.

Several unique individual patterns in physiological, biomechanical, and their inter-relationships were noted with respect to the timing of the change points. We interpret this to indicate that individuals cope with fatigue differently using different psychological abilities or skills that may lead to different patterns of physiological and biomechanical responses. Thus, one should consider excessive willingness and overconformity (44), coping strategies (45), and subconscious processes (17), as impactful factors upon the complex phenomenon of fatigue and its associated processes.

#### Effects at the change point

Finally, to gain insight into the subjective and objective changes at each of the detected change points, z-transformed scores were obtained for each variable. This provides valuable information about parameters that may be appropriate to indicate processes leading to fatigue in each individual. In this study, changes in the *motion quality*, the *breathing rate slope*, and *fatigue* were observed as indicators of the onset of fatigue. However, these parameters showed individual patterns and thus highlight the importance of an individualized approach.

### Merged consideration on overall and individual effects

The approach of capturing change points helped to uncover trends that group-wide analyses would have missed. Without the individual approach, the main conclusion would be that only subjective data are sensitive to physical load in alpine skiing; nevertheless, subjective data responded to continued alpine skiing in nine out of sixteen participants. The perceived sensitivity to fatigue-related processes in more than half of the participants supports the notion that focusing on self-awareness and self-regulation may be a beneficial approach to manage prolonged physical stress. Furthermore, individual analyses showed that changes related to the physical task elicited different individual responses; for example, two participants experienced a change in the *motion quality score*, and three in the *breathing rate slope*. This indicates that individual responses must be viewed in a very detailed manner. Thus, we emphasize the importance of a holistic, intra-individual approach utilizing subjective and objective parameters when examining fatigue processes.

## Limitations

The findings of this study must be considered with several limitations. First, the sample size of 22 participants was reduced to 16 by the strict inclusion criteria regarding minimum number of runs completed and a definite change in fatigue. The number of participants from which valid physiological and biomechanical data were collected was only 12 and 10, respectively. Thus, the sample sizes are very small and differ between data levels. Therefore, it is essential to expand to these findings with more skiers and accurate data by using more sophisticated data processing, for example, with machine learning. Second, this study was conducted with experienced male alpine skiers and the findings are therefore limited to this specific group. Third, the alpine skiing session included to perform 80 turns per minute with the best skiing technique. This was introduced to standardize the physical load, with the goal of being totally fatigued after 10 runs; it is quite different from unconstrained recreational skiing. Fourth, the analyses are limited to specific parameters measurable with available wearable sensor technology.

## Conclusion

This study showed that with the response to prolonged physical stress is highly individual. Research on fatigue in alpine skiing is still in its infancy, and little is known about inter- and intra-individual changes in the sensation of fatigue associated with perceived fatigability and performance fatigability. A more precise knowledge of the underlying processes triggered by the physical stress of alpine skiing could possibly contribute to a reduced risk of skiing accidents. However, future research needs to clarify how fatigue processes affect action errors and how this is causally related to recreational skiing accidents. Focusing on self-regulation and self-awareness may play a key role, as subjective variables are generally and in most of the individuals of this study sensitive to the physical and mental stress in alpine skiing. In the future, customized algorithms could be developed that provide feedback on ongoing fatigue processes based on sensitive parameters on the subjective, physiological, and biomechanical levels. This tool could help to train the self-awareness of fatigue in alpine skiing and thus improve the self-regulation of skiers, which in turn could have a positive effect on the risk of action errors.

## Data availability statement

The original contributions presented in the study are included in the article/Supplementary materials, further inquiries can be directed to the corresponding author/s.

## Ethics statement

The studies involving human participants were reviewed and approved by Ethics Committee of the Paris Lodron University Salzburg. The participants provided their written informed consent to participate in this study.

## Author contributions

TF, GA, and SW: development of the research design. TF: writing—original draft preparation and visualization. TB: data collection, data processing, and statistical analyses. SK: change point detection. EH: processing of breathing parameters. CS: processing of biomechanical data. SW: statistical consulting. GA: responsibility for the entire project. All authors contributed to the article and approved the submitted version.

## Funding

This work was partly funded by the Austrian Federal Ministry for Transport, Innovation and Technology, the Austrian Federal Ministry for Digital and Economic Affairs, and the federal state of Salzburg under the research program COMET—Competence Centers for Excellent Technologies—in the project Digital Motion in Sports, Fitness and Well-being (DiMo). Special thanks to Planai-Hochwurzen-Bahnen GmbH, who provided the lift tickets free of charge. The Planai-Hochwurzen-Bahnen GmbH was not involved in the study design, collection, analysis, interpretation of data, the writing of this article, or the decision to submit it for publication.

## Conflict of interest

Author CS was employed by company Red Bull Athlete Performance Center. The remaining authors declare that the research was conducted in the absence of any commercial or financial relationships that could be construed as a potential conflict of interest.

## Publisher's note

All claims expressed in this article are solely those of the authors and do not necessarily represent those of their affiliated organizations, or those of the publisher, the editors and the reviewers. Any product that may be evaluated in this article, or claim that may be made by its manufacturer, is not guaranteed or endorsed by the publisher.
