# Supplementary material for: Effects of physical stress in alpine skiing on psychological, physiological, and biomechanical parameters: An individual approach
Source: Front Sports Act Living. 2022 Oct 10;4:971137. doi: 10.3389/fspor.2022.971137 (PMC9589513; doi:10.3389/fspor.2022.971137)
Supplement: Supplementary file 1 [file Data_Sheet_1.PDF]

Supplementary Material

Table 1. *Descriptive Statistics, Skewness and Kurtosis for subjective variables (n = 16)*

	Run	*M*	*SD*	*Mdn*	Range	Skew.	Kurt.
vitality	2	8.38	1.36	8.50	6-10	-0.38	2.06
	3	7.81	1.47	8.00	5-10	-0.32	2.24
	4	7.63	1.63	8.00	4-10	-0.42	2.72
	5	7.44	2.10	7.00	4-10	-0.07	1.89
	6	7.63	1.75	8.00	5-10	-0.18	1.93
	7	6.63	1.89	7.00	4-10	0.07	1.98
	8	6.44	2.22	6.50	2-10	-0.12	2.53
	9	6.06	2.14	6.00	2-10	-0.21	2.64
	10	5.94	2.54	7.00	1-10	-0.43	2.28
fatigue	2	3.94	1.84	4.50	1-7	-0.17	1.91
	3	4.13	1.78	4.00	1-7	0.10	2.13
	4	4.81	1.80	4.50	2-7	-0.14	1.68
	5	5.44	2.13	5.00	2-8	-0.25	1.85
	6	5.19	1.94	5.50	2-8	-0.27	2.02
	7	6.31	1.92	6.50	3-9	-0.34	2.11
	8	6.69	1.99	6.50	3-9	-0.18	1.85

	9	7.25	1.77	7.50	4-9	-0.54	2.10
	10	8.06	1.81	8.50	5-10	-0.38	1.82
worry	2	1.94	1.73	1.50	0-5	0.50	2.04
	3	2.19	1.87	2.00	0-6	0.67	2.41
	4	2.63	2.28	2.50	0-7	0.41	2.03
	5	3.13	2.50	2.50	0-8	0.46	2.18
	6	3.44	2.37	3.00	0-8	0.36	2.29
	7	4.56	2.56	4.00	1-9	0.14	1.82
	8	4.63	2.63	4.00	0-9	0.10	1.94
	9	5.63	2.83	5.00	2-10	0.23	1.61
	10	5.81	3.27	6.00	0-10	-0.24	1.89
calm	2	7.25	1.84	7.00	4-10	-0.31	2.37
	3	6.81	2.40	7.00	2-10	-0.46	2.44
	4	6.63	2.28	7.00	3-10	-0.18	2.42
	5	6.63	2.60	7.50	2-10	-0.39	2.15
	6	6.94	2.14	7.00	3-10	0.08	2.01
	7	6.06	2.49	7.00	2-10	-0.15	2.16
	8	5.75	2.41	6.00	1-10	0.07	2.68
	9	5.81	2.64	6.00	1-10	-0.11	2.20
	10	5.94	2.72	6.50	0-10	-0.43	2.82

Note. Kurt. = kurtosis; Skew. = skewness

Table 2. *Descriptive Statistics, Skewness and Kurtosis for psychophysiological variables (n = 12)*

	Run	*M*	*SD*	*Mdn*	Range	Skew.	Kurt.
Breathing rate, mean [bpm]	2	55.46	11.20	53.93	35-71	-0.14	2.00
	3	56.84	11.59	53.43	37-79	0.30	2.47
	4	56.17	12.68	52.92	42-80	0.58	1.98
	5	57.96	11.24	53.69	46-84	1.13	3.36
	6	55.89	10.74	55.06	41-74	0.23	1.82
	7	56.99	12.33	55.61	41-81	0.48	2.33
	8	58.93	10.85	58.29	45-78	0.37	1.97
	9	58.27	10.16	57.06	45-78	0.48	2.30
	10	58.86	10.10	57.95	40-76	-0.02	2.42
Breathing rate, cv [%]	2	15.93	5.85	15.90	7.8-25.3	0.17	1.89
	3	17.05	5.25	15.06	9.0-24.7	0.21	1.79
	4	19.05	8.71	18.04	8.2-40.0	1.04	3.83
	5	19.96	7.50	18.43	10.9-35.2	0.48	2.34
	6	18.26	7.03	18.66	6.2-30.6	-0.09	2.41
	7	19.23	5.94	16.83	11.4-30.7	0.65	2.21
	8	19.16	6.25	16.20	12.8-33.0	1.02	2.91
	9	19.65	6.80	18.02	10.0-31.8	0.18	1.94
	10	19.05	5.85	18.09	11.8-28.4	0.39	1.86

Breathing rate, slope	2	0.10	0.07	0.10	0.02-0.25	0.58	2.45
	3	0.09	0.05	0.08	0.04-0.17	0.60	2.00
	4	0.07	0.07	0.07	-0.06-0.19	-0.03	2.38
	5	0.07	0.09	0.08	-0.15-0.23	-1.00	4.94
	6	0.08	0.05	0.08	-0.01-0.16	-0.22	2.34
	7	0.05	0.05	0.06	-0.04-0.14	-0.14	2.72
	8	0.09	0.07	0.10	0.00-0.23	0.71	3.14
	9	0.07	0.04	0.08	0.00-0.14	-0.70	2.26
	10	0.07	0.06	0.08	-0.02-0.18	0.08	1.90
Breathing depth amplitude, mean [au]	2	0.0096	0.0015	0.0098	0.0075-0.0122	0.37	2.45
	3	0.0095	0.0015	0.0096	0.0070-0.0124	0.11	2.69
	4	0.0099	0.0015	0.0096	0.0070-0.0126	0.14	3.12
	5	0.0094	0.0014	0.0094	0.0072-0.0120	0.30	2.29
	6	0.0095	0.0014	0.0096	0.0070-0.0120	0.04	2.41
	7	0.0092	0.0016	0.0090	0.0071-0.0119	0.29	2.06
	8	0.0094	0.0014	0.0092	0.0077-0.0125	0.86	3.08
	9	0.0092	0.0012	0.0095	0.0075-0.0113	0.03	2.25
	10	0.0093	0.0012	0.0092	0.0069-0.0110	-0.24	2.29

Note. au = arbitrary units; cv = coefficient of variation; Kurt. = kurtosis; Skew. = skewness

Table 3. *Descriptive Statistics, Skewness and Kurtosis for biomechanical parameters (n = 10)*

	Run	*M*	*SD*	*Mdn*	Range	Skew.	Kurt.
Turn duration, mean [s]	2	0.81	0.07	0.80	0.74-0.95	1.02	2.77
	3	0.79	0.07	0.75	0.73-0.94	1.20	3.30
	4	0.79	0.08	0.77	0.67-0.95	0.76	3.46
	5	0.78	0.07	0.76	0.69-0.94	1.31	4.59
	6	0.81	0.09	0.76	0.74-0.98	1.13	2.66
	7	0.81	0.10	0.76	0.72-1.04	1.38	3.79
	8	0.79	0.08	0.75	0.69-0.95	0.88	2.88
	9	0.79	0.06	0.78	0.73-0.88	0.16	1.40
	10	0.81	0.08	0.77	0.75-1.01	1.51	4.26
Turn duration, SD [s]	2	0.15	0.09	0.13	0.06-0.38	2.02	6.27
	3	0.14	0.14	0.10	0.04-0.52	2.36	7.21
	4	0.14	0.09	0.11	0.06-0.36	1.72	4.92
	5	0.13	0.12	0.09	0.05-0.43	1.92	5.57
	6	0.20	0.19	0.11	0.08-0.64	1.64	4.08
	7	0.14	0.10	0.11	0.04-0.41	2.07	6.30
	8	0.13	0.11	0.11	0.03-0.44	2.10	6.36
	9	0.15	0.07	0.13	0.07-0.27	0.72	2.27
	10	0.19	0.17	0.13	0.08-0.65	2.25	6.63

Supplementary Material - Load management in alpine skiing

Edge angle symmetry, mean [°]	2	3.92	1.45	3.63	2.60-7.38	1.44	4.29
	3	4.07	1.79	4.11	1.87-6.90	0.46	2.03
	4	4.01	2.09	3.36	2.35-9.47	1.98	5.89
	5	3.20	0.65	3.16	2.24-4.40	0.42	2.41
	6	4.51	2.93	3.85	1.82-12.45	2.21	6.81
	7	5.15	3.67	3.74	2.80-14.89	2.11	6.22
	8	4.45	2.03	3.56	2.50-8.65	1.00	2.76
	9	3.85	1.31	3.67	1.94-6.54	0.60	2.96
	10	4.49	1.82	3.97	2.52-7.93	0.70	2.21
Edge angle symmetry, SD [°]	2	1.90	0.81	1.76	1.30-4.04	2.00	6.02
	3	2.02	1.02	2.02	0.72-3.79	0.51	2.26
	4	2.00	0.94	1.70	1.13-4.39	1.77	5.25
	5	1.48	0.53	1.33	0.83-2.42	0.49	1.95
	6	2.16	1.32	1.72	0.68-5.34	1.45	4.49
	7	2.08	1.22	1.81	0.96-5.09	1.63	4.77
	8	2.18	1.10	1.74	1.17-4.23	0.99	2.43
	9	1.89	0.76	1.85	0.87-3.69	1.12	4.33
	10	2.13	0.91	1.98	0.99-4.17	1.04	3.52
Radial force, mean [g]	2	0.65	0.09	0.62	0.56-0.84	0.98	3.04
	3	0.64	0.07	0.66	0.51-0.77	-0.16	3.34
	4	0.64	0.07	0.65	0.54-0.75	-0.04	1.75

	5	0.62	0.06	0.60	0.55-0.73	0.63	2.02
	6	0.62	0.05	0.63	0.50-0.67	-1.59	4.92
	7	0.64	0.06	0.65	0.57-0.74	0.10	2.01
	8	0.62	0.06	0.63	0.51-0.70	-0.61	2.80
	9	0.62	0.06	0.61	0.53-0.72	0.26	2.56
	10	0.60	0.07	0.60	0.49-0.71	-0.07	2.14
Radial force, SD [g]	2	0.22	0.07	0.20	0.15-0.39	1.34	4.12
	3	0.21	0.03	0.20	0.16-0.25	-0.10	2.28
	4	0.20	0.04	0.20	0.16-0.26	0.23	1.52
	5	0.20	0.03	0.20	0.14-0.26	-0.07	3.58
	6	0.21	0.04	0.22	0.14-0.27	-0.39	3.19
	7	0.20	0.04	0.20	0.15-0.25	-0.12	1.86
	8	0.21	0.08	0.18	0.11-0.39	1.22	3.95
	9	0.20	0.03	0.20	0.16-0.25	0.60	2.61
	10	0.21	0.03	0.21	0.16-0.26	-0.40	3.19
Motion quality score, mean	2	6.07	1.11	5.65	4.73-7.97	0.50	2.01
	3	5.50	0.85	5.55	4.17-6.90	-0.07	2.30
	4	5.90	1.13	5.70	4.13-7.83	0.37	2.33
	5	5.79	1.26	5.82	3.60-8.03	0.19	2.75
	6	5.48	1.12	5.60	3.67-7.30	-0.34	2.55
	7	6.22	1.40	6.05	4.40-8.53	0.30	1.80

	8	5.56	0.78	5.45	4.33-6.90	0.07	2.20
	9	5.57	0.97	5.48	4.33-7.47	0.56	2.55
	10	5.26	0.94	5.17	3.97-7.00	0.37	2.41
Motion quality score, SD	2	1.24	0.37	1.28	0.74-1.78	0.03	1.65
	3	1.35	0.41	1.30	0.87-1.90	0.12	1.41
	4	1.30	0.48	1.33	0.53-2.09	-0.19	2.27
	5	1.13	0.20	1.12	0.86-1.43	0.15	1.53
	6	1.25	0.33	1.29	0.73-1.64	-0.30	1.86
	7	1.27	0.29	1.31	0.77-1.70	-0.17	2.10
	8	1.33	0.30	1.26	0.94-1.79	0.27	1.86
	9	1.22	0.22	1.21	0.88-1.67	0.47	2.83
	10	1.30	0.31	1.23	0.92-1.76	0.45	1.76

Note. Kurt. = kurtosis; Skew. = skewness

Table 4. *Friedman's ANOVA for the remaining biomechanical parameters*

	Friedman' s ANOVA		
	χ^2	*p*	*W*
Biomechanical parameters (n = 10)			
Turn duration, mean [s]	8.15	1.0†	0.10
Edge angle symmetry, SD [°]	5.55	1.0†	0.07
Radial force, SD [g]	3.52	1.0†	0.04
Motion quality score, SD	6.71	1.0†	0.08

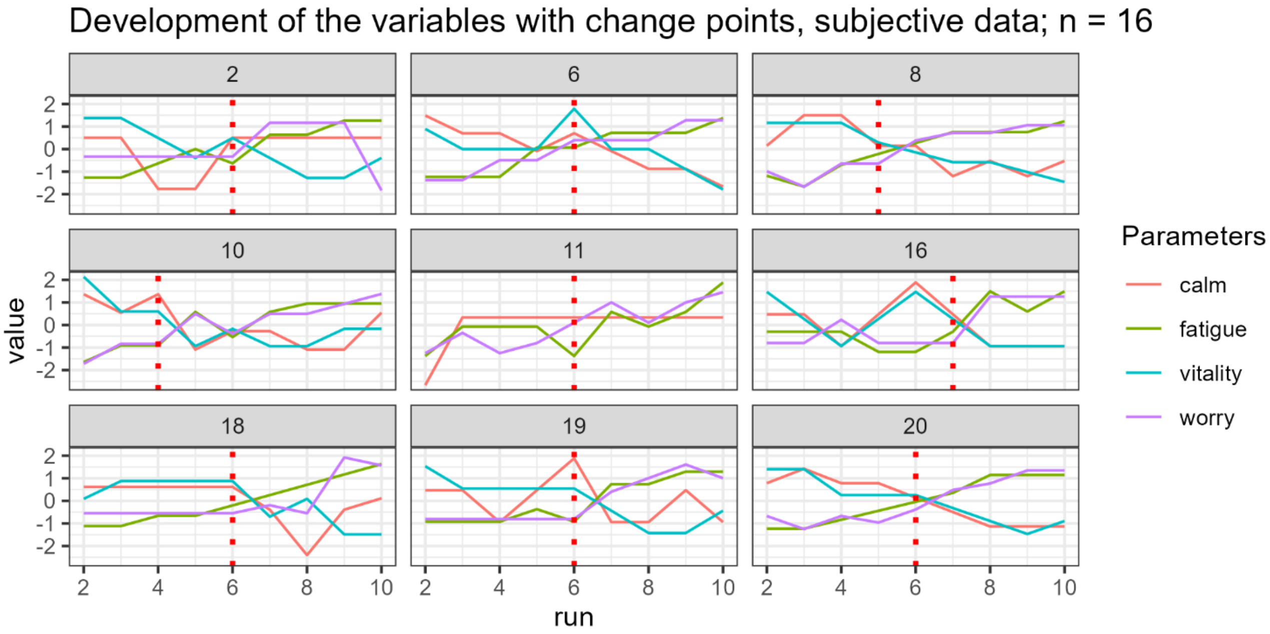

Figure 1. Z-transformed changes of subjective data including the time of the change point (vertical line)

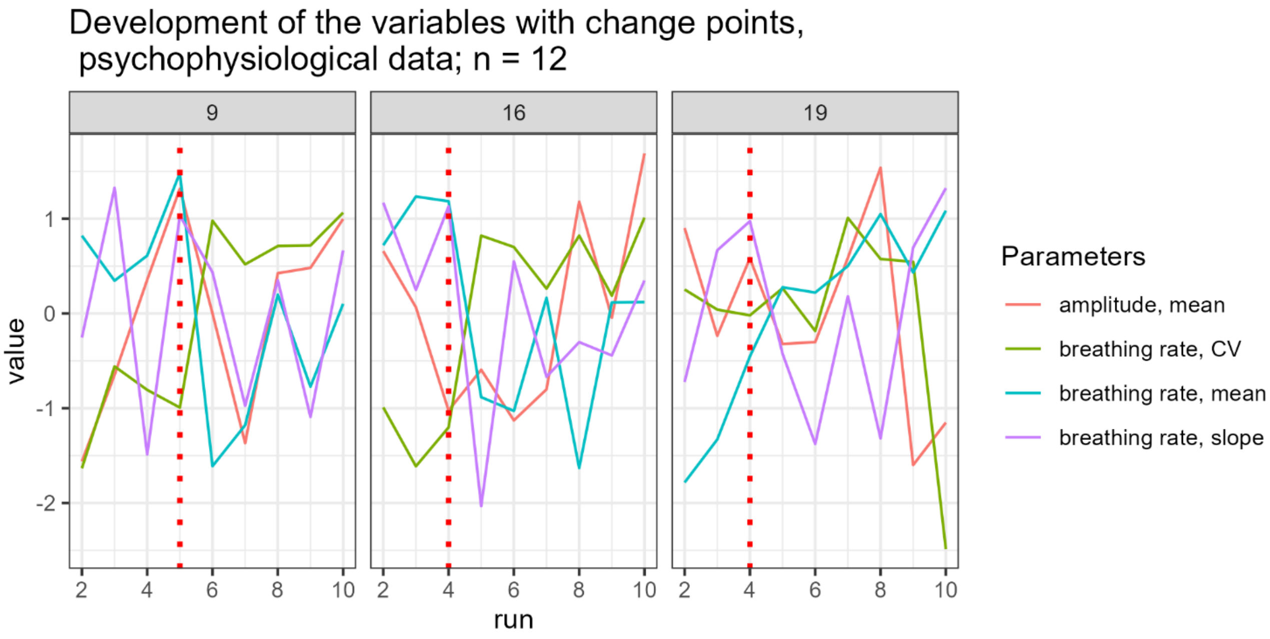

Figure 2. Z-transformed changes of physiological data including the time of the change point (vertical line)

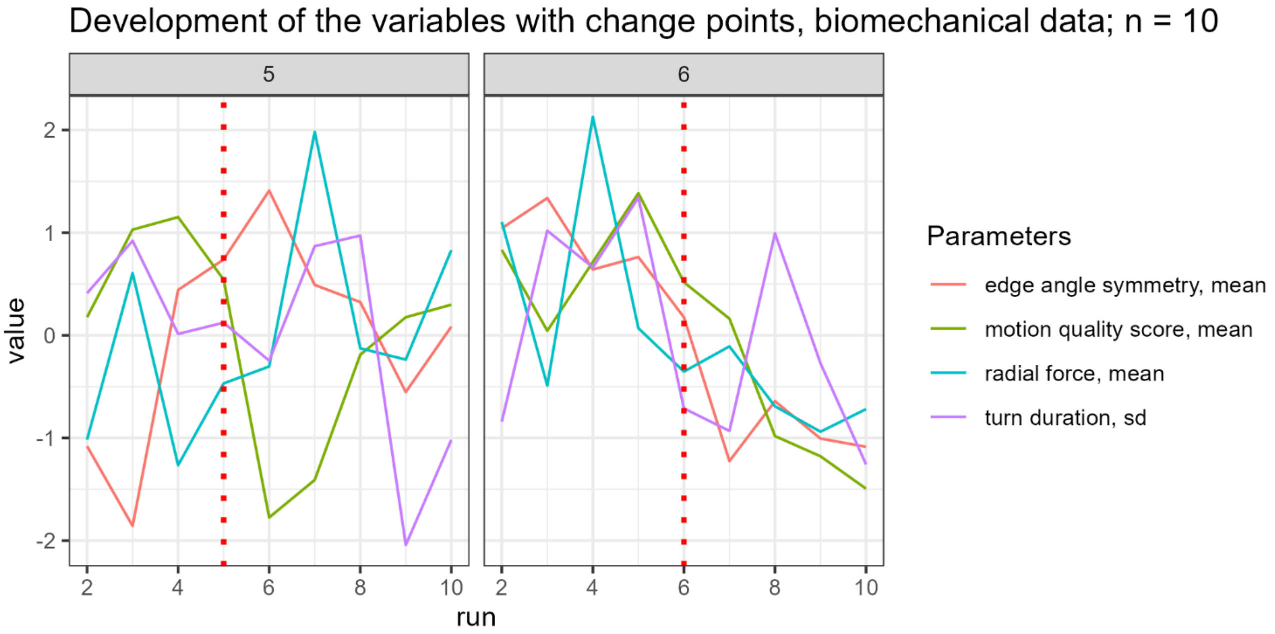

Figure 3. Z-transformed of biomechanical data including the time of the change point (vertical line)

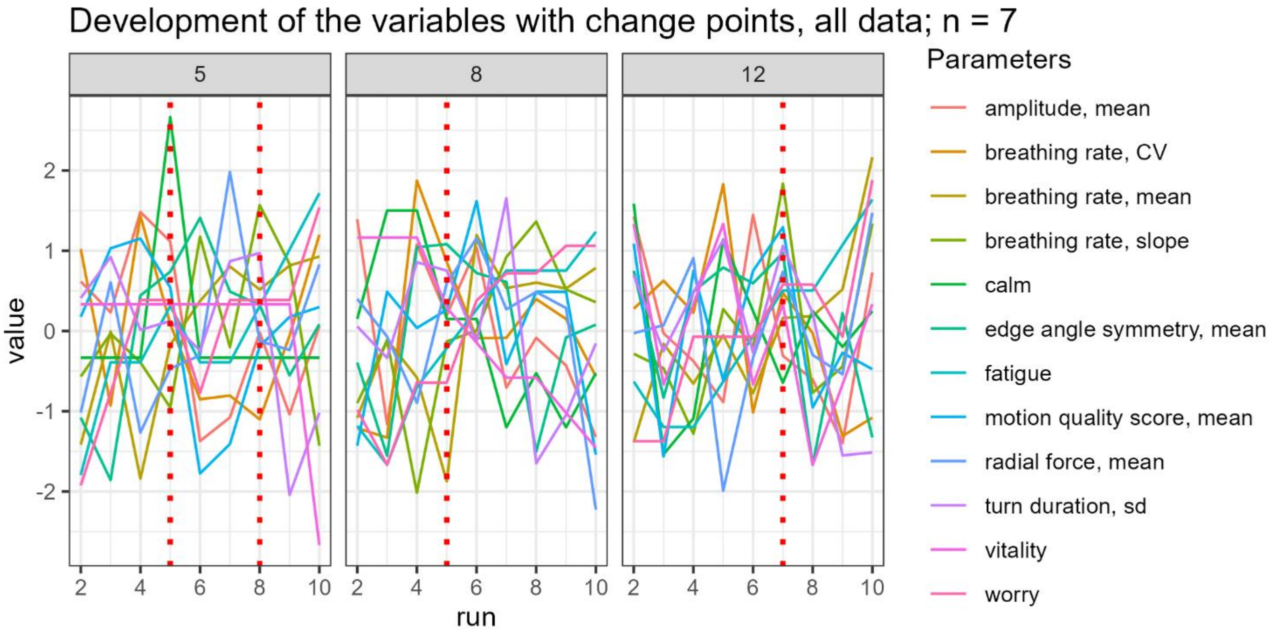

Figure 4. Z-transformed of all data including the time of the change point (vertical line)
