# Supplementary figures and images for: Effects of physical stress in alpine skiing on psychological, physiological, and biomechanical parameters: An individual approach
Source: Front Sports Act Living. 2022 Oct 10;4:971137. doi: 10.3389/fspor.2022.971137 (PMC9589513; doi:10.3389/fspor.2022.971137)

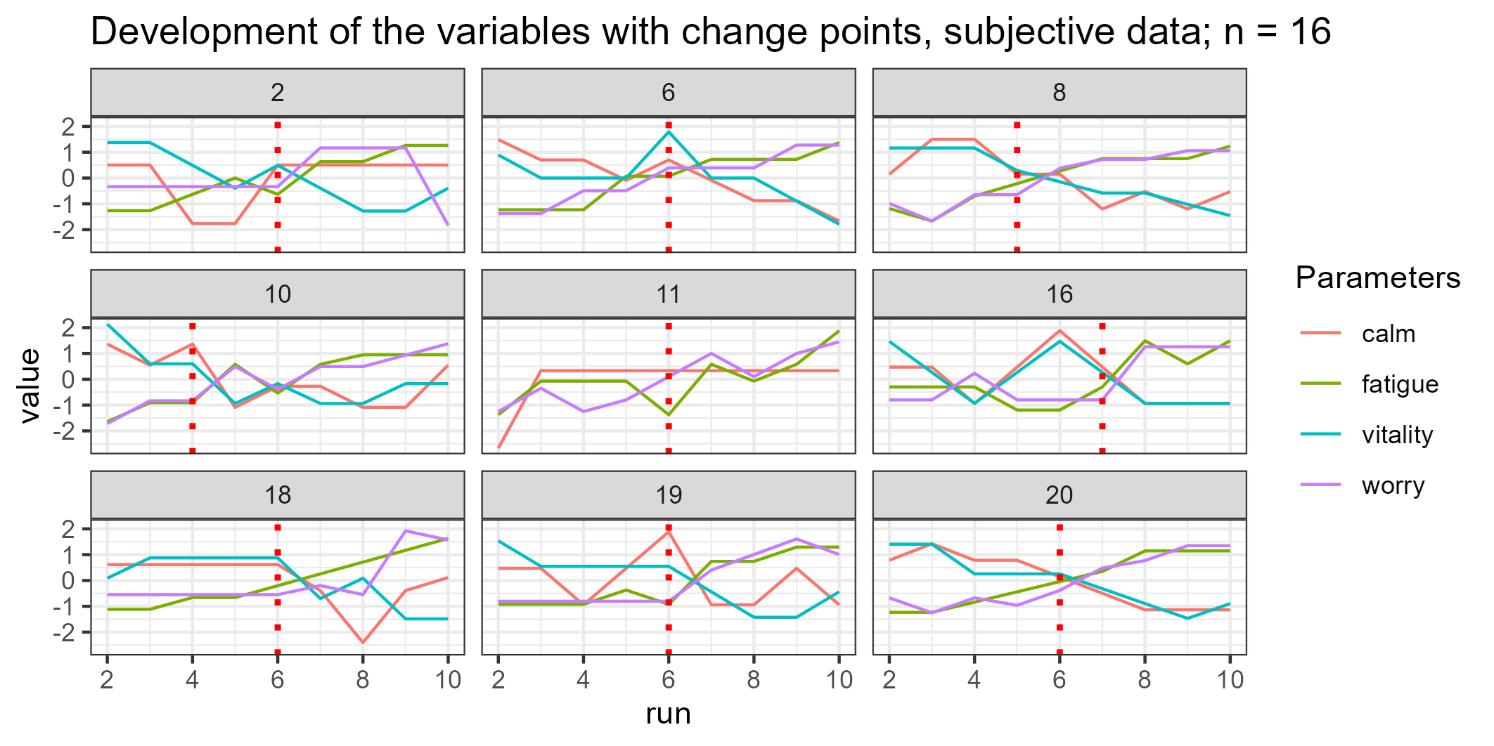

Supplement: Supplementary file 2 [file Image_1.PNG]

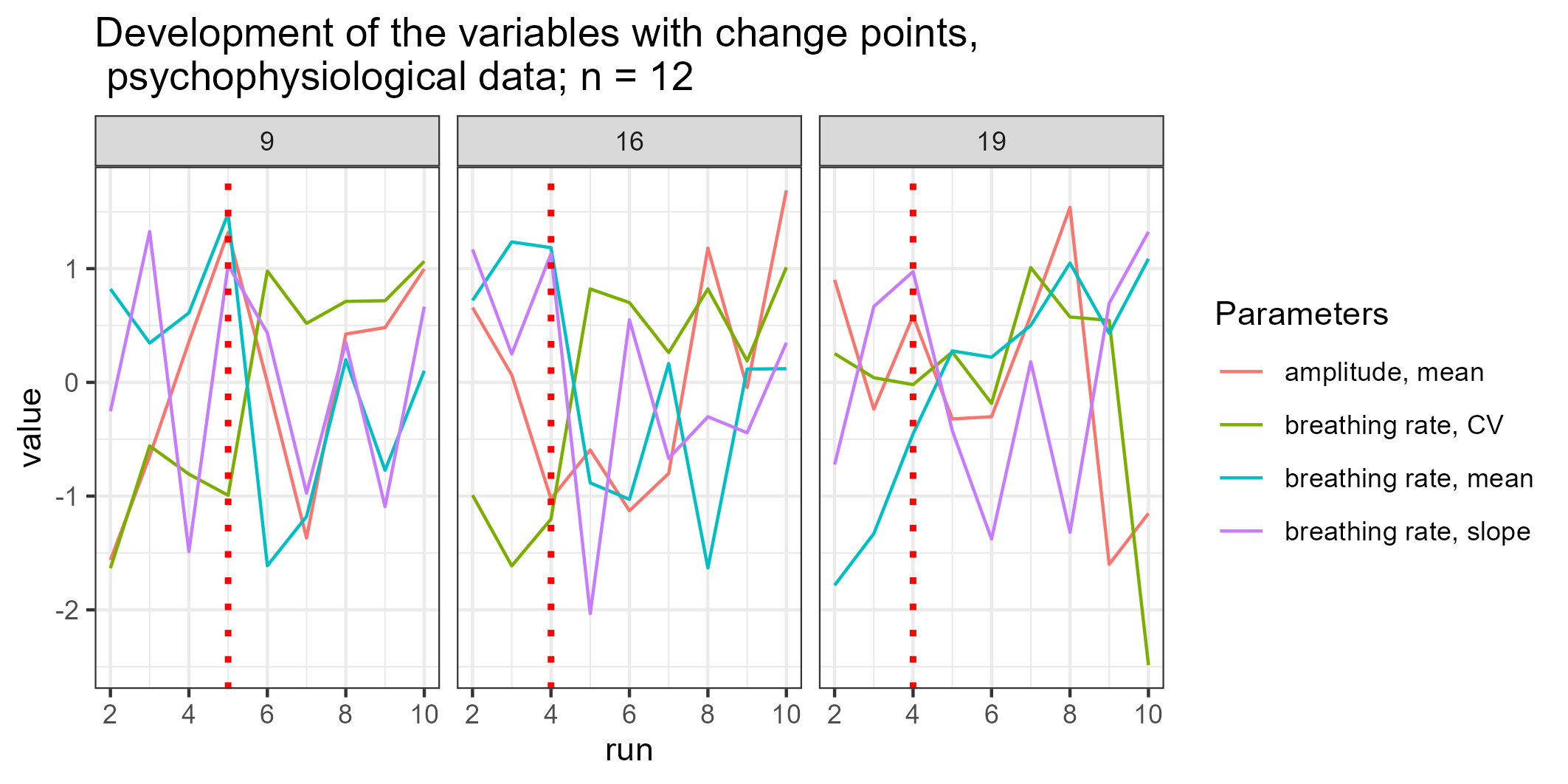

Supplement: Supplementary file 3 [file Image_2.PNG]

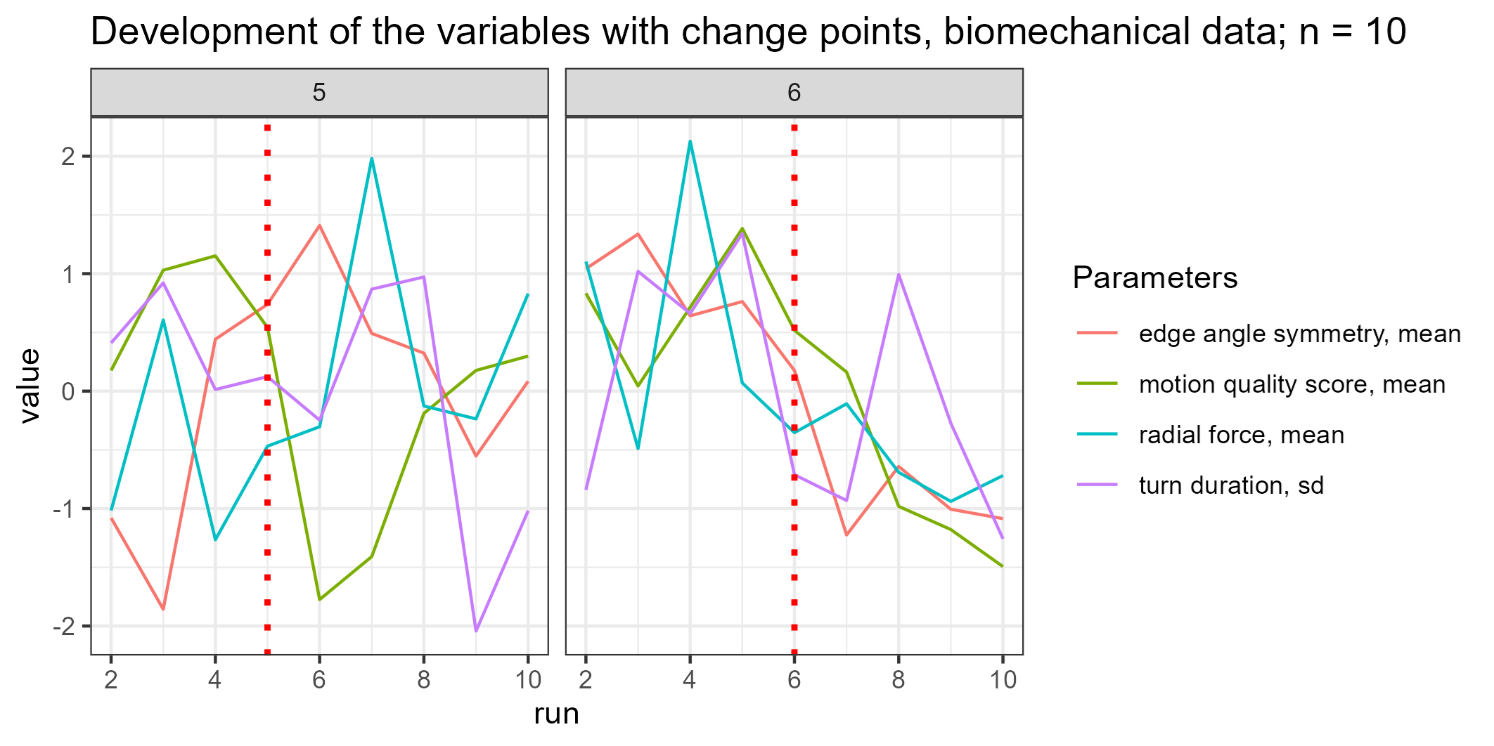

Supplement: Supplementary file 4 [file Image_3.PNG]

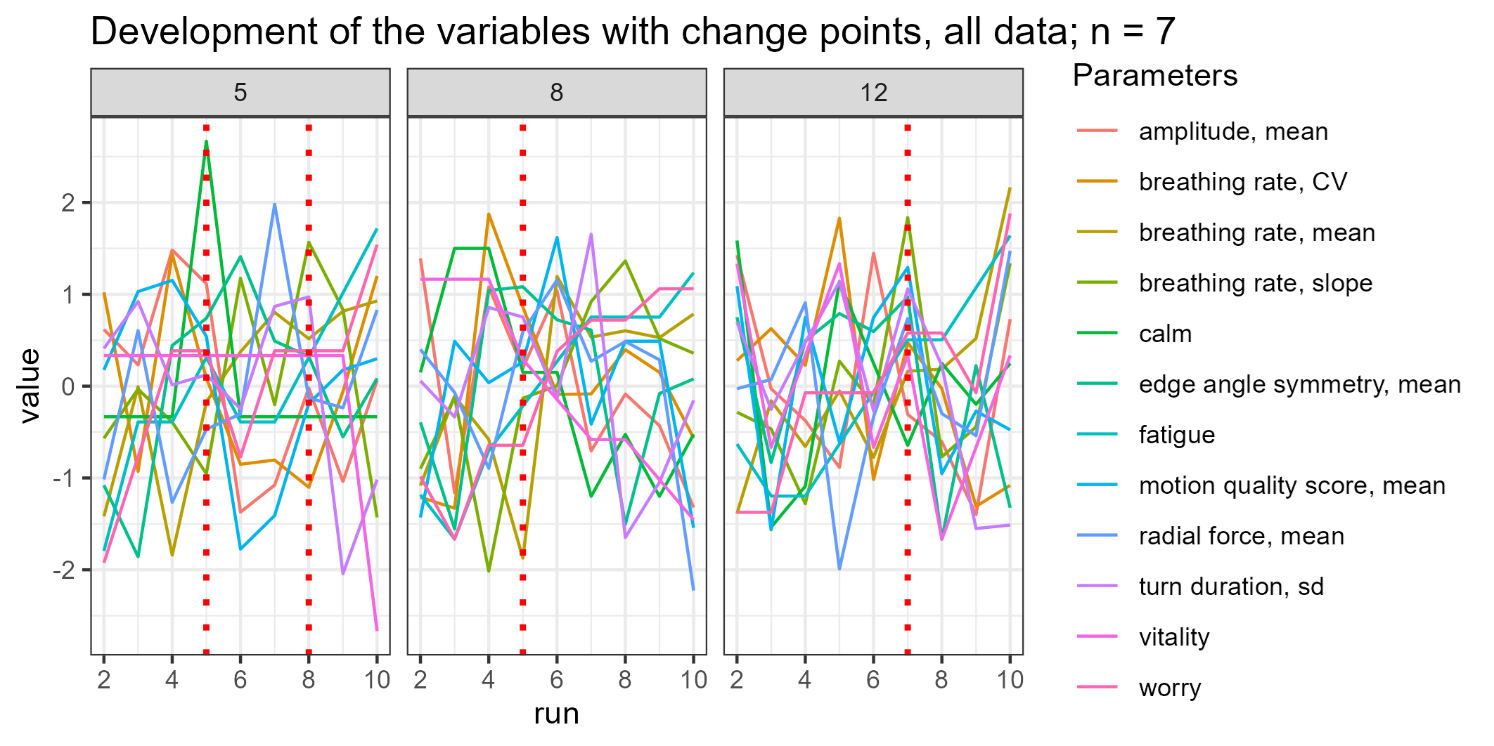

Supplement: Supplementary file 5 [file Image_4.PNG]
